# Bioinformatics analysis of tumor-educated platelet microRNAs in patients with hepatocellular carcinoma

**DOI:** 10.1042/BSR20211420

**Published:** 2021-12-08

**Authors:** Beibei Zhu, Shanshan Gu, Xiaoting Wu, Wenyong He, Hongke Zhou

**Affiliations:** Department of Gastroenterology, The First Affiliated Hospital of Jinan University, Jinan University, Guangzhou 510632, China

**Keywords:** bioinformatics analysis, hepatocellular carcinoma, microRNA, tumor-educated platelets

## Abstract

**Background:** Hepatocellular carcinoma (HCC) is one of the most prevalent malignancies that seriously threaten global health. The primary reason for its grim prognosis is the lack of sensitive tools for early diagnosis. The purpose of the present study was to apply bioinformatics analysis to explore tumor-educated platelet (TEP) microRNA (miRNA) expression and its potential diagnostic utility in HCC.

**Methods:** Twenty-five HCC patients and 25 healthy controls were included. RNA sequencing was utilized to screen miRNA alterations in platelets derived from HCC patients (*n*=5) and controls (*n*=5). Gene set enrichment analysis was performed to analyze the targeted mRNAs of differentially expressed miRNAs by using the Gene Ontology (GO) and the Kyoto Encyclopedia of Genes and Genomes (KEGG) databases, aiming at main functions and pathways, respectively. We then verified the selected platelet miRNAs in another cohort by quantitative reverse transcription-polymerase chain reaction (qRT-PCR) amplification.

**Results:** A total of 250 differentially expressed miRNAs were identified, among which 111 were down-regulated and 139 were up-regulated. The functional enrichment analysis of differentially expressed miRNAs suggested that their target genes were involved primarily in pathways related to HCC. Expression levels of miR-495-3p and miR-1293 were further validated by qRT-PCR, which yielded results consistent with the sequencing analysis. The area under the receiver operating characteristic (ROC) curve of miR-495-3p and miR-1293 as diagnostic tests for HCC were 0.76 and 0.78, respectively.

**Conclusion:** TEP miRNAs such as miR-495-3p and miR-1293 were differentially expressed in HCC patients, and may be involved in the pathophysiology of HCC.

## Introduction

Hepatocellular carcinoma (HCC) is one of the most prevalent cancers worldwide, with approximately 906000 incident cases during 2020. Due to the limited capacity for early diagnosis, HCC is one of the most deadly cancers, ranking third in cancer-related mortalities [1]. Most patients have reached an advanced stage when first diagnosed. Currently, early diagnosis of HCC relies primarily on serum tumor markers, imageology, and tissue biopsy [2]. However, these techniques lack sensitivity and specificity for early-stage diagnosis. Liquid biopsy, considered a minimally invasive, safe, and sensitive technique, facilitates the analysis of several blood-based biomarkers that may be applied for screening and early diagnosis of cancer [3,4].

Circulating platelets are anucleate cells derived from bone marrow megakaryocytes. Their primary physiological role is the regulation of hemostasis and thrombosis [5]. The effects of platelets on HCC have received increasing attention in recent decades. Platelets secrete a variety of immune mediators, which may contribute to the progression of HCC by altering the immune microenvironment [6]. Thrombocytosis is associated with the formation of tumor thrombi and an increased risk of distant metastases [7,8]. Significant reductions in the morbidity and mortality of HCC have been observed in patients treated with aspirin [9,10]. Platelets can take up tumor-derived secreted membrane vesicles that contain tumor-associated (mutant) RNAs [11]. These platelets are known as tumor-educated platelets (TEPs) [12]. Platelets can also induce specific splicing of their pre-mRNAs in response to signals from cancer cells and the tumor microenvironment, thus changing their transcriptomes, molecular profiles, and subsequent disease progression [13]. Interactions between circulating platelets and malignant cells may affect tumor growth and dissemination [12,14–16].

MicroRNAs (miRNAs) are a class of short non-coding RNAs that contain 20–25 nucleotides. Because platelets are anucleate cells, the majority of RNA transcripts and proteins are acquired from megakaryocytes and are capable of maintaining function when activated [17]. Hundreds of miRNAs have been identified in platelets [18]. However, their involvement in the development and progression of HCC, as well as their diagnostic utility, is yet to be determined. The purpose of the present study was to elucidate differentially expressed levels of TEP-derived miRNAs in HCC patients and healthy individuals, as well as to further explore platelet–tumor interactions, and to identify new biomarkers to facilitate the early clinical diagnosis and treatment of HCC.

## Materials and methods

### Inclusion of HCC patients and healthy individuals

A total of 25 HCC patients and 25 age- and gender-matched healthy individuals (controls) were selected at the First Affiliated Hospital of Jinan University from June 2019 to November 2020. Controls had no history of malignant tumor and no laboratory test abnormalities, and were matched with HCC patients by gender and age. Inclusion criteria of HCC subjects included: age over 18 years, diagnosis of primary HCC by imaging techniques or tissue biopsy, and no history of thrombosis, blood transfusion, anti-platelet medication exposures, hemorrhage, or hematologic diseases. The study protocol was approved by the Ethics Committee of the First Affiliated Hospital of Jinan University (approval number: KY-2020-046), and written informed consent was obtained from each participant.

### Leukocyte-depleted platelet isolation and total platelet RNA extraction

Aliquots of 5 ml blood were collected from each participant in anticoagulant tubes containing ethylene diamine tetra acetic acid. Blood samples were diluted with PBS (1:1 ratio) containing 10% sodium citrate (v/v) and layered on a density gradient (Ficoll, Thermo Fisher, U.S.A.). We then centrifuged the samples at 400×***g*** for 35 min at 20°C to obtain mononuclear layers. Another centrifugation at 300×***g*** was then performed for 10 min to obtain platelet suspensions from supernatants. Leukocyte-depleted platelets (LDPs) were pelleted at 300×***g*** for 10 min and then resuspended in 40 μl buffer, which was prepared by diluting MACS BSA stock solution 1:20 with autoMACS rinsing solution. Human CD61 MicroBeads reagent was used to purify platelets, which were added to the platelet suspension and mixed gently. After incubation for 15 min, a magnetic separating unit was used to deplete CD61-positive cells. The remaining LDPs were collected and lysed in lysis/binding buffer, after which total platelet RNA was harvested with the miRNeasy Mini Kit (QIAGEN GmbH, Hilden, Germany) according to the manufacturer’s instructions. A NanoDrop spectrophotometer (Thermo Scientific, Utah, U.S.A.) was used to measure the concentration and purity of total RNA, which was stored at −80°C until further processing.

### Construction of gene library and sequencing data analysis

Five cases were randomly selected from each group for sequencing analysis. The extracted RNA was applied to prepare miRNA-focused next-generation sequencing libraries using a QIAseq miRNA library kit (QIAGEN, catalog number: 331505, Germantown, MD, U.S.A.), according to the manufacturer’s instructions. Sequencing was performed on an Illumina Hiseq 2500 instrument of HaploX Biotechnology Co., Ltd. (Shenzhen, China). Data analysis was performed using a QIAseq miRNA quantitative platform and unique molecular index (UMI) counting. Raw data were normalized. Fold change (FC) indicated the ratio of miRNA expression between two groups. miRNAs with *P*-values ≤0.05 and | log2FC | >2 were considered to be differentially expressed. R (version 3.5.1) statistical language and R Studio software were used for bioinformatics analysis. Differentially expressed miRNA target genes were predicted by TargetScan (http://www.targetscan.org), miRwalk, miRDB (http://www.mirdb.org/index.html), and PicTar software (some miRNAs can only be found in three softwares, three prevailed). Gene set enrichment analysis was performed to analyze the targeted genes of differentially expressed miRNAs by using the Gene Ontology (GO) and Kyoto Encyclopedia of Genes and Genomes (KEGG) databases, aiming at main functions and pathways, respectively.

### Expression analysis by quantitative real time polymerase chain reaction

Five miRNAs exhibiting up- or down-regulated expression were chosen for result verification by RT-PCR in all 40 samples (including the samples used in sequencing analysis). Aliquots of 2 µg total RNA were used to perform reverse-transcription using the PrimeScript™ RT Reagent Kit with gDNA Eraser (RR047A, TAKARA, Dalian, China). Q-PCR was performed using TB Green™ Premix ExTaq™II (RR820A, TAKARA, Dalian, China) in the Bio-Rad CFX96 real-time PCR detection system (Bio-Rad). Spiked-in *cel-miR-39* was used as the internal control. Relative miRNA expression levels were calculated by the 2^−ΔΔ*c*_t_^ method. Primer sequences are shown in Supplementary Table S1.

### Analysis of The Cancer Genome Atlas dataset

To verify our findings in an independent dataset, another HCC cohort from The Cancer Genome Atlas (TCGA) database was included and analyzed. This TCGA cohort has miRNA detected in 359 tumor tissues and 50 adjacent normal tissue samples, whose normalized miRNA levels were obtained from http://gdac.broadinstitute.org. Related clinical information including TNM staging, overall survival, and survival status were obtained from cBioportal (https://www.cbioportal.org).

### Standardized processing of sequencing data and statistical analysis

To standardize the original data, HTSeq-count software was used to calculate gene expression levels, eliminate reads with count values <10, and to obtain gene expression count matrices for downstream analysis. The original count data were filtered by counts per million to remove genes that were not expressed in most samples. Principal component analysis was used to investigate relationships between samples. Additionally, a receiver operating characteristic (ROC) curve was calculated to evaluate the power of differentially expressed miRNAs in HCC diagnosis. Pearson’s chi-squared test or Fisher’s exact test was used to compare differences of characteristic features. Expression levels of certain genes as evaluated by quantitative reverse transcription-polymerase chain reaction (qRT-PCR) were denoted as mean ± SD, and differences were evaluated by *t* tests. *P*<0.05 and *P*<0.001 were considered significant. All statistics were performed and presented using GraphPad Prism 8 (GraphPad Software Inc., La Jolla, CA, U.S.A.).

## Results

### Clinical and laboratory characteristics

Both the HCC and control groups consisted of 25 persons each, with the same gender ratio (14 men and 6 women) (Table 1). Serum levels of alanine aminotransferase and albumin in HCC patients were lower than those of controls. According to Barcelona Clinic Liver Cancer staging, 3 patients had HCC at the very early stage, 9 were at early stage, 2 were in the intermediate stage, and 6 had late-stage disease. Portal vein tumor thrombosis was found in 4 patients (20%).

**Table 1:** Characteristics of five patients and five controls

Variables	HCC (*n*=25)	HC (*n*=25)	*P*-value
Age (years)*	51.3 ± 10.93	52.95 ± 10.23	0.31
Gender (women/men)	6/14	6/14	
Platelet count (×10^9^/l)*	162.3 ± 54.09	179 ± 63.3	0.18
ALT (U/l)*	67.9 (27.75, 78.75)	24 ± 9.33	*P*<0.001
Albumin (g/l)*	50.52 ± 19.75	41.75 ± 4.39	*P*<0.05
Total bilirubin (μmol/l)*	25.69 (13.9, 27.28)	14.7 ± 4.05	0.16
AFP (ng/ml)	2622 (6.99, 2853)	-	
Hepatitis B virus infection (%)	18/20 (90%)	-	
Liver cirrhosis (%)	17/20 (85%)	-	
PVTT (%)	4/20 (20%)	-	
BCLC stage (0/A/B/C/D)	3/9/2/6	-	

Values are presented as frequency (percent), mean ± standard deviation (SD) or mean (95% CI), where appropriate. *Difference between HCC group and HC group is calculated by Chi-square test or Student’s *t* test, where appropriate, and *P-*value <0.05 is considered statistically significant. Abbreviations: AFP, α fetoprotein; ALT, alanine aminotransferase; BCLC, Barcelona Clinic Liver Cancer; HC, healthy control; PVTT, portal vein tumor thrombus.

### Identification of differentially expressed miRNAs in HCC

**Figure 1:**
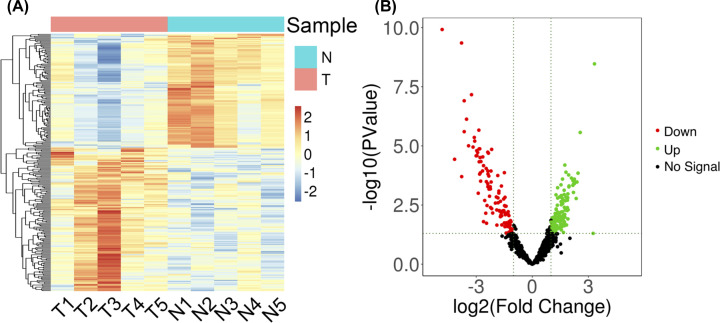
Differentially expressed miRNAs in HCC patients vs healthy controls detected with RNA-seq (**A**) Heatmap demonstrating genes that were differentially expressed between platelets derived from HCC patients and controls. (**B**) Volcanic map showing differential gene expression. Genes with corrected *P*-value <0.05 and |log2FC| >1 were defined as differentially expressed genes. N, normal; T, tumor.

The median gene expression values of each sample were similar after standardization of the level of counts values. Principal component analysis showed large differences between the two groups (Supplementary Figure S1). A total of 250 differentially expressed miRNAs were screened as conditions, of which 111 miRNAs were down-regulated and 139 were up-regulated. The heatmap and volcano map of differential genes are shown in Figure 1. Table 2 shows the top ten up- and down-regulated miRNAs.

**Table 2:** Top ten significant up- and down-regulated differentially expressed miRNAs

Down-regulated	Up-regulated				
Gene	LogFC	*P*-value	Gene	LogFC	*P*-value
*miR-495-3p*	−4.820	1.20E-10	miR-4634	3.326	1.14E-06
*miR-337-3p*	−4.154	3.68E-05	miR-7108-3p	2.564	3.02E-04
*miR-136-5p*	−3.782	4.46E-10	miR-1293	2.447	3.87E-03
*miR-379-3p*	−3.775	0.000199	miR-7150	2.390	0.006515
*miR-376b-3p*	−3.640	1.24E-07	miR-6832-5p	2.256	7.41E-03
*miR-337-5p*	−3.515	7.42E-07	miR-8077	2.250	5.56E-03
*miR-412-5p*	−3.249	6.84E-08	miR-8056	2.239	6.30E-03
*miR-487b-3p*	−3.166	1.12E-05	miR-4443	2.176	6.29E-03
*miR-1185-1-3p*	−3.100	4.39E-06	miR-6722-3p	2.094	5.26E-03
*miR-539-3p*	−3.077	6.28E-06	miR-3182	1.945	4.26E-03

The top ten differentially expressed miRNAs were ranked according to the value of |log FC| between two groups. LogFC means the ratio of expression between two groups. *P<*0.05 is considered to be significant.

### Functional analysis of differentially expressed miRNAs

**Figure 2:**
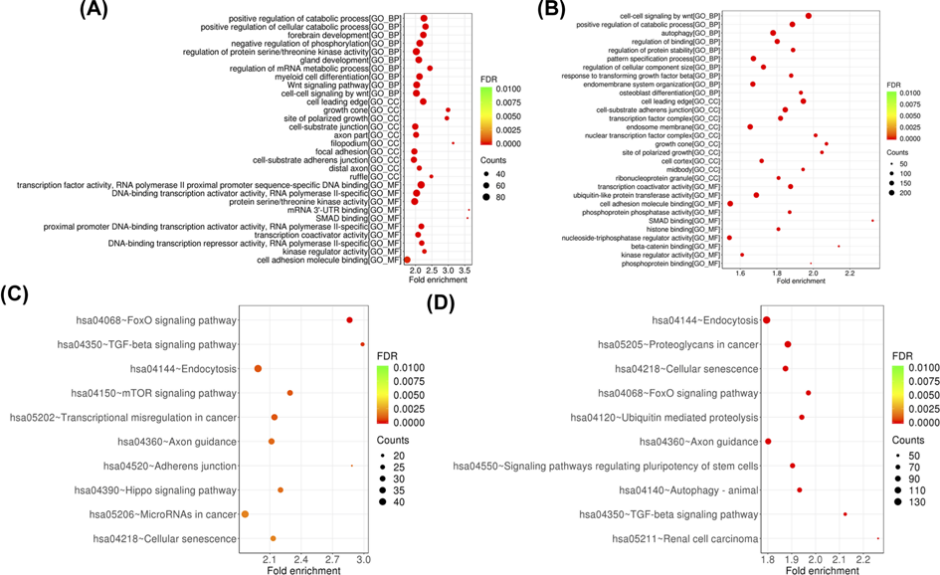
Functional analysis of differentially expressed miRNAs in GO and KEGG databases (**A**) Top ten fold-enrichment molecular functions, biological processes, and cellular components of up-regulated miRNAs. (**B**) Top ten fold-enrichment molecular functions, biological processes, and cellular components of down-regulated miRNAs. (**C**) Top ten fold-enrichment pathways of up-regulated miRNAs. (**D**) Ten-fold enrichment pathway of down-regulated miRNAs.

GO and KEGG pathway analyses were performed on the target gene of miRNAs that were differentially expressed in HCC to determine their potential biological functions. The GO (biological process, molecular functions, and cellular components) and KEGG pathways of up-regulated (Figure 2A,C) and down-regulated (Figure 2B,D) miRNAs in HCC were analyzed separately. Top ten fold-enrichment function are shown in Figure 2. Differentially expressed miRNAs target genes were involved in the positional regulation of catabolism and modulation of serine/threonine protein kinase activity, mRNA metabolism, and Wnt pathway signaling, most of which are closely related to tumor metabolism. KEGG pathway analysis also showed that platelet miRNAs are involved in multiple signaling pathways, including FoxO, ubiquitin-mediated proteolysis, axon guidance, autophagy-animal, TGF-β signaling pathways, and mTOR signaling pathways.

### Validation and diagnostic value of miRNAs

**Figure 3:**
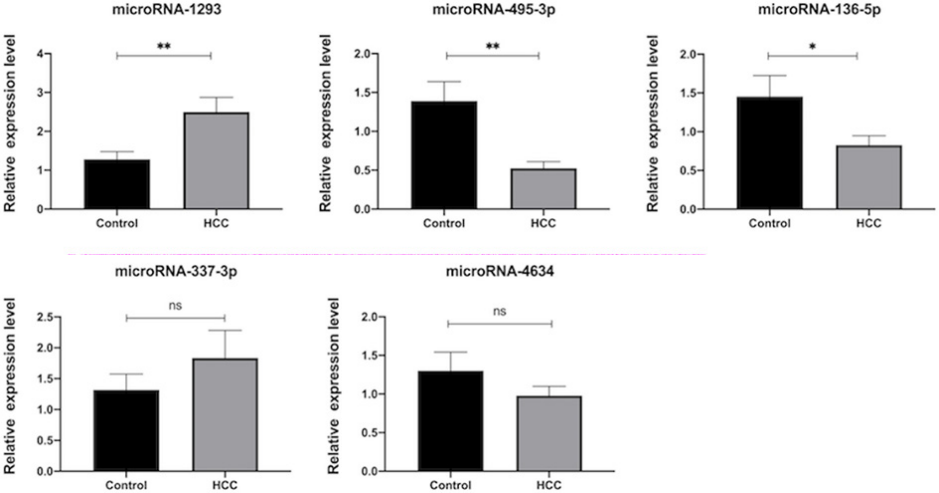
RT-qPCR validation of differentially expressed miRNAs in HCC and control groups (**A**) miR-1293, (**B**) miR-495-3p, (**C**) miR-136-5p, (**D**) miR-337-3p, (**E**) miR-4634. Significance: *P-value <0.05, **P-value <0.01.

**Figure 4:**
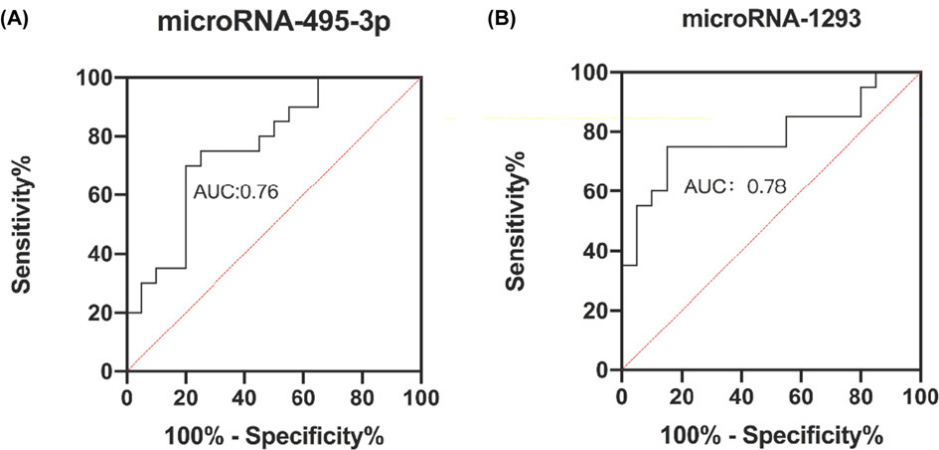
ROC curve for the evaluation of the accuracy of differentially expressed miRNAs to discriminate patients with HCC from healthy controls (**A**) The AUC of miR-495-3p was 0.76. (**B**) The AUC of miR-1293 was 0.78.

To verify our observations, we confirmed the expression of five up-regulated or down-regulated miRNAs (miR-495-3p, miR-337-3p, miR-136-5p, miR-1293, and miR-4634) by qRT-PCR. Compared with controls, the expression level of miR-495-3p and miR-136-5p were significantly lower in HCC patients, while the level of miR-1293 held an oppositely higher expression (Figure 3). Furthermore, the areas under the ROC curves (AUCs) for miR-1293 and miR-495-3p were 0.78 and 0.76, respectively (*P*<0.05; Figure 4).

**Figure 5:**
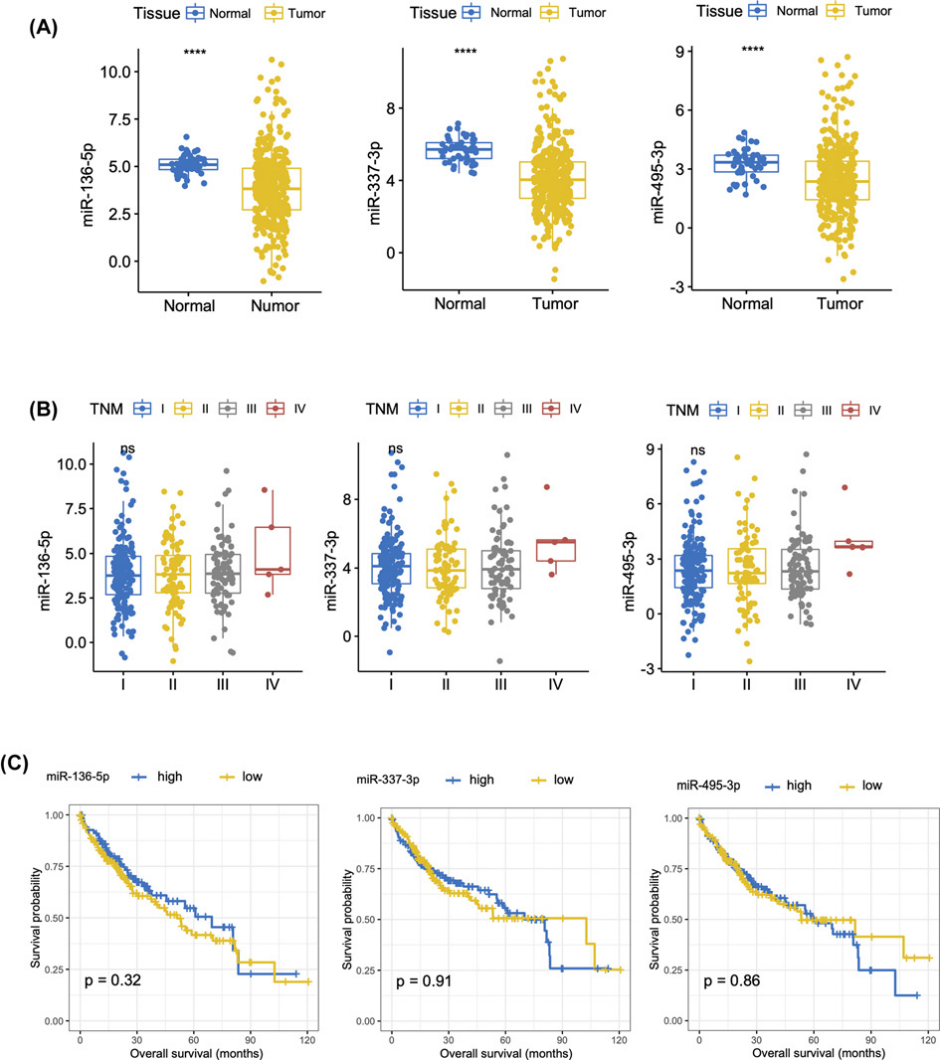
TEP miRNA DE expression and association with HCC stage and survival based on TGCA database (**A**) Expression levels of miR-136-5p, miR-495-3p miR-337-3p in normal and HCC tissues. (**B**) Association between miR-136-5p, miR-495-3p, and miR-337-3p and HCC stage. (**C**) The association between miR-136-5p, miR-495-3p, and miR-337-3p and survival months. ****P-value <0.0001.

To further validate the clinical significance of the miRNAs identified in our study, we evaluated the clinical relevance of these miRNAs using the HCC dataset of the TCGA database. Of note, miRNAs detected in the TCGA cohort were derived from tumor tissue rather than circulating platelets. As for the five differentially expressed miRNAs identified in the present study (miR-495-3p, miR-337-3p, miR-136-5p, miR-1293, and miR-4634), three (miR-495-3p, miR-337-3p, miR-136-5p) have data from all patients in the TCGA cohort and thus were analyzed here. We found that all three miRNAs were down-regulated in tumor tissues (Figure 5A), but demonstrated no differential expression across different TNM stages (Figure 5B). None were associated with overall survival (Figure 5C).

## Discussion

New targets and strategies are urgently needed to broaden the limited diagnostic and treatment options for HCC. Accumulating evidence has revealed multiple roles of TEP miRNAs in various pathophysiological processes as well as cancer pathogenesis [16]. TEPs can react to tumor growth and treatment and identify specific tumor types [19]. Although miRNAs are the most studied small RNAs in the pathogeneses of numerous human diseases, relatively little is known about their involvement in platelet biology.

In the present study, we performed bioinformatics analysis to clarify differential platelet miRNA expressions between patients with HCC and healthy individuals. Compared with controls, 250 differentially expressed miRNAs were identified in the platelets of HCC patients, among which 111 were significantly down-regulated and 139 were up-regulated. qRT-PCR results revealed that miR-495-3p and miR-136-5p were significantly lower, while miR-1293 was higher in the platelets of HCC patients, which verified our sequencing results. In addition, the analyses of potential biological functions indicated that the up-regulated miRNAs took part in cellular catabolism. The Wnt-β catenin signaling pathway is hyperactivated in HCC and promotes tumor growth and dissemination [20]. Dysregulation of the transforming growth factor β signaling pathway plays a central role in inflammation, fibrogenesis, and immunomodulation in the HCC microenvironment. Down-regulated miRNAs affect the response to transforming growth factor β, which may represent a biomarker to formulate individualized immunotherapies for HCC [21]. Wu et al*.* [22] found that Lnc RNA LUCAT1 may affect autophagy in HCC through the hsa-miR-495-3p/DLC1 axis. Collectively, our results showed that the differentially expressed miRNAs target genes are involved in various biological processes.

MiR-495-3p represents a potential therapeutic target in clinical oncology. miR-495 underexpression has been associated with esophageal squamous cell carcinoma [23]. Functional loss or suppression of miR-495-3p triggers transformation and growth of cancer cells [24]. Down-regulated miR-136-5p was observed in HCC tissue compared with normal tissue. Ding et al. [25] demonstrated that TNM stage, vasoinvasion, and metastasis were negatively associated with miR-136-5p expression. miR-136-5p may serve a crucial function in the pathogenesis and development of HCC via the regulation of transcription, enzyme-linked receptor protein signaling, macromolecule metabolism, and several important pathways of carcinogenesis and progression, including the MAPK and p53 signaling pathways. *In vitro*, miR-136 (miR-136-5p) was down-regulated by hepatitis B virus X protein. Decreased miR-136-5p expression can up-regulate astrocyte elevated gene-1 to promote cell migration [26]. miR-136-5p may act as a tumor suppressive miRNA in various cancers. Down-regulated miR-136-5p expression has been demonstrated in epithelial ovarian cancer and glioma cell lines [27,28]. miR-136 (miR-136-5p) expression was decreased in metastatic compared with non-metastatic giant cell tumor of bone [29]. miR-136 (miR-136-5p) inhibited mobility, invasiveness, and epithelial–mesenchymal transition in lung cancer cells by targeting Smad2 and Smad3 [30].

Correlations between miR-1293 expression and the clinicopathological features and prognosis of HCC remain unclear. Phosphatidylinositol-3-kinase (PI3K-AKT) has been recognized as an important candidate in different tumors as molecular features as well as a classic dysregulated pathway involved in the pathogenesis of HCC. The PI3K-AKT pathway was reported to be regulated by miR-1293, which explains the involvement of miR-1293 in the HCC [31]. Subsequent investigation found that miR-1293 suppresses tumor cell growth by targeting BRD4 and DNA repair genes at the same time. miR-1293 could be a promising target for the development of miRNA-based cancer therapeutics. Further investigation is required to evaluate the clinical relevance of miR-1293 [32].

Even with these promising findings, several limitations of our study must be addressed. First of all, the sample size of our study was small. Secondly, our study did not analyze associations between TEP miRNA levels in HCC patients and tumor characteristics, such as tumor stage, microvascular invasion, and differentiation. Future studies with larger sample sizes are required to confirm our results regarding the associations of platelet miRNAs and HCC. Further research is required to explore whether these TEP miRNAs may be used to develop clinical diagnostic strategies and to guide precision therapy.

In conclusion, we found that TEP miRNAs were differentially expressed in HCC patients compared with healthy controls. Bioinformatics analysis showed that differentially expressed miRNAs may participate in the metabolism, differentiation, adherence, and other physiological processes of tumor cells. Our findings may play an essential role in elucidating HCC tumorigenesis and progression.

## Supplementary Material

Supplementary Figure S1 and Table S1

## Data Availability

The raw data in the present study can be found in GEO datasets (link: http://www.ncbi.nlm.nih.gov/geo) with the accession number GSE158523.

## Abbreviations

FC: fold change
GO: Gene Ontology
HCC: hepatocellular carcinoma
KEGG: Kyoto Encyclopedia of Genes and Genomes
LDP: leukocyte-depleted platelet
miRNA: microRNA
PI3K: phosphatidylinositol-3-kinase
qRT-PCR: quantitative reverse transcription-polymerase chain reaction
ROC: receiver operating characteristic
TCGA: The Cancer Genome Atlas
TEP: tumor-educated platelet
