# Supplementary material for: Bioinformatics analysis of tumor-educated platelet microRNAs in patients with hepatocellular carcinoma
Source: Biosci Rep. 2021 Dec 8;41(12):BSR20211420. doi: 10.1042/BSR20211420 (PMC8661502; doi:10.1042/BSR20211420)
Supplement: Supplementary Figure S1 and Table S1 [file BSR-2021-1420_supp.pdf]

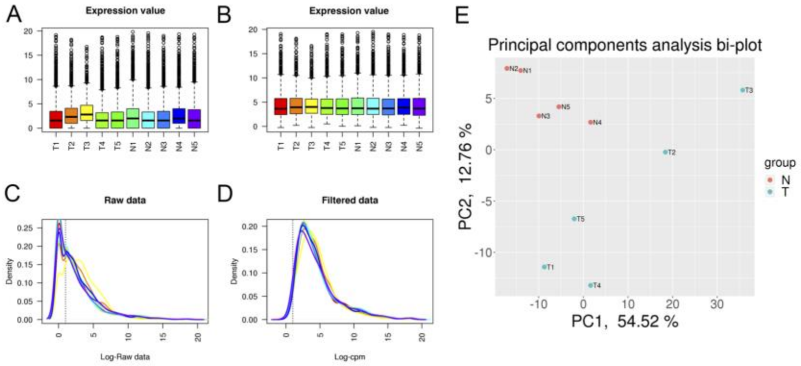

Figure S1. The standardization of sequencing data. (A) Original read count value boxplot. (B) Normalized read count value boxplot. (C) Original read count value density map. (D) Normalized read count value density map. (E) Principal component analysis of standardized read count values of two groups of samples, N for healthy controls and T for HCC group.

Table S1. qRT-PCR primer sequences used in this study

Gene name		Nucleotide sequence
has-miR-495-3p	Forward	5'-ACAAACATGGTGCACTTCTT-3'
	Reverse	5'-GTGCAGGGTCCGAGGT-3'
has-miR-337-3p	Forward	5'-CCUAUAUGAUGCCUUUCUUC-3'
	Reverse	5'-GTGCAGGGTCCGAGGT-3'
has-miR-4634	Forward	5'-CGGCGCGACCGGCCCGGGG-3'
	Reverse	5'-GTGCAGGGTCCGAGGT-3'
has-miR-1293	Forward	5'-GGTGGTCTGGAGATTTG-3'
	Reverse	5'-GAACATGTCTGCGTATCTC-3'
has-miR-136-5p	Forward	5'-CGCGACTCCATTTGTTTTGA-3'
	Reverse	5'-AGTGCAGGGTCCGAGGTATT-3'
